# Differential Diagnosis Assessment in Ambulatory Care With a Digital Health History Device: Pseudorandomized Study

**DOI:** 10.2196/56384

**Published:** 2025-10-01

**Authors:** Beth Healey, Adrien Schwitzguebel, Herve Spechbach

**Affiliations:** 1 Emergency Department University Hospitals of Geneva Geneva Switzerland; 2 AS Medicine du Sport Hopital de la Providence Neuchatel Switzerland; 3 Department of Primary Care Medicine University Hospitals of Geneva Geneva Switzerland

**Keywords:** differential diagnosis, decision-making, artificial intelligence, machine learning, digital diagnosis, computer-assisted, digital assessments, hospital-outpatient clinics, emergency, clinical applications software, diagnosis, ambulatory care, digital health, pseudorandomized study, digital tools, digital technology, medical consultation, decision support, health informatics

## Abstract

**Background:**

Digital health history devices represent a promising wave of digital tools with the potential to enhance the quality and efficiency of medical consultations. They achieve this by providing physicians with standardized, high-quality patient history summaries and facilitating the development of differential diagnoses (DDs) before consultation, while also engaging patients in the diagnostic process.

**Objective:**

This study evaluates the efficacy of one such digital health history device, diagnosis and anamnesis (DIANNA), in assisting with the formulation of appropriate DDs in an outpatient setting.

**Methods:**

A pseudorandomized controlled trial was conducted with 101 patients seeking care at the University Hospital Geneva emergency outpatient department. Participants presented with various conditions affecting the limbs, back, and chest. The first 51 patients were assigned to the control group, while the subsequent 50 formed the intervention group. In the control group, physicians developed DD lists based on traditional history-taking and clinical examination. In the intervention group, physicians reviewed DIANNA-generated DD reports before interacting with the patient. In both groups, a senior physician independently formulated a DD list, serving as the gold standard for comparison.

**Results:**

The study findings indicate that DIANNA use was associated with a notable improvement in DD accuracy (mean 79.3%, SD 24%) compared with the control group (mean 70.5%, SD 33%; *P*=.01). Subgroup analysis revealed variations in effectiveness based on case complexity: low-complexity cases (1-2 possible DDs) showed 8% improvement in the intervention group (*P*=.08), intermediate-complexity cases (3 possible DDs) showed 17% improvement (*P*=.03), and high-complexity cases (4-5 possible DDs) showed 15% improvement (*P*=.92). The intervention was not superior to the control in low-complexity cases (*P*=.08) or high-complexity cases (*P*=.92). Overall, DIANNA successfully determined appropriate DDs in 81.6% of cases, and physicians reported that it helped establish the correct DD in 26% of cases.

**Conclusions:**

The study suggests that DIANNA has the potential to support physicians in formulating more precise DDs, particularly in intermediate-complexity cases. However, its effectiveness varied by case complexity and further validation is needed to assess its full clinical impact. These findings highlight the potential role of digital health history devices such as DIANNA in improving clinical decision-making and diagnostic accuracy in medical practice.

**Trial Registration:**

ClinicalTrials.gov NCT03901495; https://clinicaltrials.gov/study/NCT03901495

## Introduction

In modern health care, digital health history devices (DHHDs) are playing an increasingly significant role in improving the quality and efficiency of medical consultations. These devices offer standardized patient history summaries, which have the potential to enable more accurate differential diagnoses (DDs) by gathering comprehensive patient data before medical consultations. Research has demonstrated that DHHDs enhance clinical decision-making by reducing diagnostic errors and improving efficiency, allowing health care providers to focus on critical aspects of care rather than repetitive information gathering [1,2]. By streamlining the data acquisition process, DHHDs facilitate more informed decision-making, benefiting both patients and health care professionals [3].

Among these devices is “DIANNA” (diagnosis and anamnesis), which has undergone substantial improvements since its initial development. A previous randomized controlled trial provided early insights into the tool’s impact on diagnostic accuracy and efficiency. Building on this foundation, DIANNA now includes a body pictogram feature to better select symptomatic areas, making it more intuitive and comprehensive for clinicians [4,5]. This enhanced version of DIANNA is designed to bridge the gap between patient-reported symptoms and clinical assessment, offering clearer data for health care professionals with the aim of improving the diagnostic process. The integration of artificial intelligence (AI) within DIANNA further augments its capabilities. AI features such as natural language processing algorithms, machine learning models, and predictive analytics provide valuable diagnostic support and assist health care professionals by suggesting DDs based on patient data [6,7].

Despite the promising advancements in DHHDs, significant gaps remain in the literature regarding their real-world efficacy across diverse clinical settings and their performance in larger patient populations. While prior studies have demonstrated that AI-driven tools can enhance diagnostic precision [8,9], research specifically focused on DHHDs such as DIANNA remains limited, particularly in assessing their long-term impact on clinical outcomes and their integration into routine practice.

This study aims to evaluate the efficacy of the updated version of DIANNA in supporting clinical diagnosis, building upon previous findings while addressing the knowledge gap surrounding its role in enhancing diagnostic accuracy and efficiency in real-world settings. By assessing its effectiveness in DD formulation, this research seeks to establish a clearer understanding of how DIANNA can assist health care professionals in making more informed clinical decisions and ultimately contribute to improving patient outcomes.

## Methods

### Study Design

This study used a single-center, unblinded, 1:1 pseudorandomized design to evaluate the efficacy of DIANNA. The first 50 patients recruited were assigned to the control group, while the subsequent 50 were allocated to the intervention group. The study did not require follow-up and no modifications were made to the protocol after the trial commenced. Throughout the trial, no significant technical or digital issues necessitated corrections.

### Patient Population

The study enrolled adult patients who sought care at the University Hospital Geneva emergency outpatient department between February 2019 and June 2020. Recruitment was conducted through a combination of methods to ensure broad participation. Patients presenting with a qualifying complaint were invited to participate by health care providers, with eligibility criteria communicated at the time of their visit. This convenience sampling approach facilitated the inclusion of a diverse patient population seeking emergency care.

To enhance awareness, the study was also advertised through flyers posted within the emergency department and internal communication with health care providers. Interested patients who met the inclusion criteria were provided with detailed information about the study’s objectives and the digitalized DIANNA tool, allowing them to voluntarily express their willingness to participate.

Patients were eligible if they presented with an injury or medical condition affecting the upper limb, trunk, or lower limb and were able to fully use DIANNA. Exclusion criteria included strictly dermatologic conditions, straightforward injuries (such as toe, hand, or ankle inversion injuries), urgent medical conditions, and patients unable to complete the DIANNA tool, including those with visual impairments, elderly individuals, and non-Francophones.

### Pseudorandomization and Recruitment

Recruitment for the study occurred in 2 phases. The first 50 patients designated as the “control group” were recruited between February 2019 and April 2019. The subsequent 50 patients allocated to the “intervention group” were recruited across 2 distinct periods: November to December 2019 and June to July 2020. The recruitment process was influenced by several factors, including the availability of the coordinating researcher and the impact of the COVID-19 pandemic. Due to pandemic-related restrictions and disruptions, recruitment was paused during certain periods and resumed when feasible. Whenever a patient was identified by the emergency department software, the coordinating researcher assessed their eligibility for enrollment, with final confirmation made by the senior physician (SP). Recruitment continued until 50 patients were enrolled in each group, ensuring balanced representation for analysis and comparison. Any adaptations to the protocol due to the pandemic, if applicable, were not mentioned in the Methods section and should be clarified for transparency.

### DIAANA Tool

DIAANA is supporting the diagnostic process by introducing a comprehensive body segmentation system with zone-selection pictograms, replacing traditional questions regarding symptom localization. This innovative approach involves an interactive questionnaire that patients complete prior to their consultation. Instead of a lengthy list of 269 questions, DIAANA uses multiple-choice formats to collect relevant data. Subsequently, the software uses AI to analyze this information and suggest a list of potential diagnoses from a pool of 126 diagnostic entities.

The AI-driven reasoning within DIAANA emulates the decision-making process of a specialist physician to establish a DD. The resulting information is then seamlessly conveyed to the physician in a user-friendly format, which includes a concise patient history summary, highlighting pertinent elements from the questionnaire. In addition, the report provides a list of possible diagnoses, completed with emergency-level categorization, potential contributing factors, and first-line management recommendations. This innovative approach not only streamlines the diagnostic process but also enhances the quality of care provided to patients.

### Intervention

In this study, 2 groups of resident physicians (RPs) were involved. The first group used the DHHD while the second group did not have access to this device. For patients in the intervention group, they were instructed to independently complete DIANNA using a touch screen tablet, guided by the coordinator, without external assistance. The DIANNA-generated summary was then printed and provided to the RP before the patient’s consultation.

After the consultation, but before reviewing additional medical test results, the RP compiled a list of potential DDs from a digital diagnosis list without external support. The case was subsequently presented to the SP, who independently selected their own DD from the same digital list to establish a “gold standard” for comparison.

**Figure 1:**
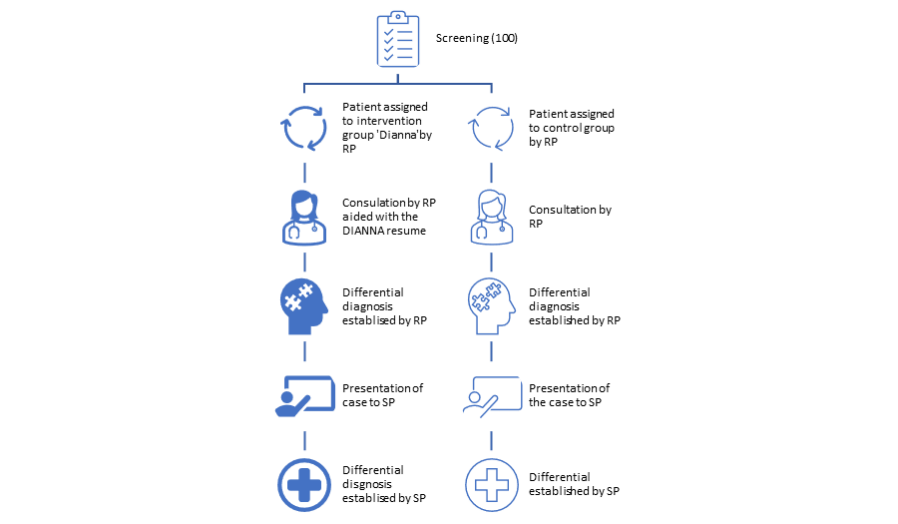
Study flowchart. RP: resident physician; SP: specialized physician.

The SP’s assessment was made without influence from the resident’s evaluation or the DHHD report, ensuring an impartial evaluation. This setup allowed for a comparison between the 2 groups, one with the aid of the DHHD and one without, to assess the impact of this technology on the diagnostic process (Figure 1).

### Outcomes

The outcomes of the study are listed in Textbox 1.

In summary, the study’s comprehensive array of outcome measures aimed to assess the impact of DIANNA on diagnostic accuracy, consultation efficiency, patient satisfaction, and the perspectives of RPs. The novel stratification of DD accuracy based on case complexity added a unique dimension to the evaluation of DHHDs’ performance in clinical practice.

**Primary and secondary outcome measures.**

**Primary outcomes**

- The percentage of accurate differential diagnoses (DDs) established by resident physicians (RPs) using diagnosis and anamnesis (DIANNA) in comparison to the reference “gold-standard” assessments conducted by senior physicians.

**Secondary outcomes**

Secondary outcome measures encompassed a range of critical aspects, including:

- Consultation time: this outcome assessed the duration of patient-physician interactions, aiming to determine if the use of DIANNA impacted the efficiency of the consultation process.
- Patient satisfaction: patient satisfaction was evaluated on multiple dimensions, including:
  Comprehensibility of questions (1-5 Likert scale): patients provided feedback on the clarity and ease of understanding the questions presented by DIANNA.
  Accuracy in describing symptoms (percentage): patients rated the precision of how DIANNA described their symptoms.
  Time taken to complete: this measure gauged the time efficiency of completing the DIANNA questionnaire.
  Relevance to presenting complaint (percentage): patients assessed the extent to which DIANNA’s questions were pertinent to their specific medical concern.
  Preference for completion location and keeping summary (percentages): patients indicated their preference for completing the questionnaire at home and retaining a copy of the DIANNA summary for their records.
- Physician resident feedback: this outcome solicited feedback from RPs and included assessments of:
  DIANNA’s contribution to suggesting DDs (percentage): RPs indicated if DIANNA provided valuable insights and suggested DDs that they might have otherwise overlooked.
  Exhaustiveness of the DD list: physicians assessed the completeness of the DD list generated by DIANNA.
  Time savings (1-5 Likert scale): physicians rated the extent to which DIANNA expedited the diagnostic process on a Likert scale.
- Interest in future use in clinical practice (percentage): physicians expressed their willingness to incorporate DIANNA into their future clinical practice.
- Percentage of correct DDs according to case complexity: this outcome measure stratified the accuracy of DDs based on the complexity of the cases. Case complexity was defined by the number of DDs present in the “gold-standard” reference DDs, categorizing them into low complexity (1-2 DDs), intermediate complexity (3 DDs), and high complexity (4-5 DDs).

### Statistical Analyses

The sample size for each group, consisting of 50 patients, was determined based on insights gained from our prior study [10]. Our rationale for this sample size calculation was as follows: we aimed to achieve 80% statistical power to detect a significant difference at the 5% level. Specifically, we sought to detect an increase in the primary outcome measure from an average of 59% correct diagnoses in the control group to 75% correct diagnoses in the experimental group. Given the study’s robust design and expected low dropout rate, we determined that a total sample size of 100 patients was appropriate for this investigation.

For data analysis, we used descriptive statistics to characterize baseline characteristics. To assess group differences, we applied appropriate statistical tests. Table 1 explicitly states the statistical tests used to compare the demographic and clinical characteristics between the groups.

We used an intention-to-treat analysis, ensuring that all patients were included in the analysis. In cases of missing data, patients were not excluded; instead, analyses were conducted on available data.

The distribution of all variables was assessed using standard graphical analyses. As the distribution was determined to be parametric, we used the Student *t* test for all comparisons.

In addition, we conducted an analysis of covariance to adjust for potential confounders. This analysis focused on the primary outcome (use of DIANNA) and considered the number of diagnoses established by the SP, as well as other baseline characteristics where a *P* value of less than .2 was identified in the univariate analysis. The following covariates were included:

1. Global analysis, low complexity, and high complexity subgroups: number of diagnoses, chief resident’s years of experience, and internal resident’s evaluation.
2. Intermediate complexity subgroup: chief resident’s years of experience and internal resident’s evaluation (excluding the number of diagnoses).

Statistical significance was defined as *P*<.05.

All analyses were conducted using R v3.4.2 Portable, provided by the Free Software Foundation Inc. This analytical approach ensured a rigorous evaluation of study outcomes and allowed us to draw meaningful conclusions from the data.

**Table 1:** Baseline characteristics.

			DIANNA (n=50)		Control group (n=51)		*P* value
Age (years), mean (SD), range			34.1 (13), 18-65		38.5 (12), 17-63		.08
Male sex, n (%)			34 (68)		36 (71)		.95
Resident physician’s practice (years), mean (SD), range			7.5 (3), 1-12		8 (3), 4-12		.36
Resident physician’s skills (1-4) (years), mean (SD), range			2.7 (1), 1-4		2.4 (1), 1-4		.09
Senior physician’s practice (years), mean (SD), range			6.9 (2), 1-11		6 (3), 1-10		.07
Senior physician’s skills (1-4) (years), mean (SD), range			2.9 (1), 1-4		2.8 (1), 1-4		.64
Consultation time (years), mean (SD), range			3.7 (2), 1-10		3.2 (2), 2-11		.28
Initial complaint, n (%)							
	Shoulder pain and trauma	5 (10)		6 (12)		>.99	
	Elbow pain	7 (14)		5 (10)		.73	
	Wrist and hand pain	9 (18)		7 (14)		.75	
	Back pain and trauma	2 (4)		5 (10)		.45	
	Pelvis pain	2 (4)		3 (6)		>.99	
	Knee pain and trauma	11 (22)		5 (10)		.16	
	Ankle trauma	4 (8)		3 (6)		.98	
	Foot trauma	3 (6)		9 (18)		.13	
	Soft tissue trauma and swelling	7 (14)		8 (16)		>.99	
Case complexity (N of DD; years), mean (SD), range			2.6 (1), (1-5)		2.3 (1), 1-5		.23
Diagnoses found by resident physician (years), mean (SD), range			0.8 (0), 0.25-1		0.7 (0), 0-1		.13

### Ethical Considerations

This study was approved by the Medical Ethics Committee of the University Hospitals Geneva in Switzerland (REQ-2017-00878). The study protocol was formally registered at ClinicalTrials.gov (NCT03901495) before recruitment commenced. Given the study’s observational nature and the absence of any investigational drug or invasive procedures, an expedited ethical review process was conducted.

Since the study posed minimal risk to participants and did not involve any interventions beyond standard clinical procedures, oral informed consent was deemed appropriate. Each participant was provided with a concise written description of the study’s aims, methods, and potential implications. The informed consent process ensured that participants understood the study and voluntarily agreed to participate before proceeding with DIANNA.

To safeguard patient privacy and maintain data confidentiality:

1. All collected data were anonymized, with no personally identifiable information linked to study records.
2. Patient responses and physician assessments were securely stored in an encrypted database accessible only to authorized research personnel.
3. The DIANNA tool operated on a secure platform without storing patient identifiers, ensuring compliance with data protection regulations.
4. Data analysis was conducted on deidentified datasets, minimizing risks of breaches in patient confidentiality.

Participants were not financially compensated for their involvement in this study, as the research was deemed noninterventional and posed no additional burden beyond routine medical care. However, participants were given the opportunity to retain a copy of their DIANNA summary for their own reference, which many found beneficial for future consultations.

## Results

### Overview

In this study, a total of 101 patients were screened and evenly divided into 2 groups: 50 in the intervention group and 51 in the control group. Importantly, no patients were lost to follow-up during the course of the study, ensuring a comprehensive dataset for analysis. The preintervention characteristics, including patient demographics, case complexity, and initial complaints, exhibited no significant differences between the 2 groups, establishing a strong baseline for comparison.

### Demographics

Of the 101 patients screened, 50 were assigned to the intervention group and 51 to the control group. All were included in the study as none were lost to follow-up. Preintervention patient demographics, case complexity, and initial complaints did not differ between the groups (Table 1).

### Differential Diagnosis

When assessing the primary outcome, the percentage of correct DDs, it was observed that the intervention group, which used DIANNA, outperformed the control group. The intervention group achieved a mean of 79.3% accuracy in DDs, with a SD of 24%, while the control group had a mean accuracy of 70.5%, with a SD of 33%. This difference was statistically significant with a *P* value of .01, highlighting the effectiveness of DIANNA in improving the accuracy of DDs.

Furthermore, the analysis considered the impact of case complexity on DD accuracy. It was found that DIANNA consistently exhibited benefits across all levels of case complexity. Specifically, for low-complexity cases (1-2 DDs possible), the intervention group displayed an 8% improvement over the control group (*P*=.08). In intermediate-complexity cases (3 DDs), the improvement was even more pronounced at 17% (*P*=.03), and for high-complexity cases (4-5 DDs), the impact was substantial, though not statistically significant, at 15% (*P*=.91).

In addition to these findings, DIANNA demonstrated an overall DD accuracy of 72% for the entire cohort, with notably higher accuracy for low-complexity cases at 88%, followed by 84% for moderate-complexity cases, and 75% for high-complexity cases.

The accuracy of the intervention was not superior in comparison to the control group for low-complexity cases (*P*=.08) and for high-complexity cases (*P*=.92; Table 2).

In Table 2, the outcome variable for the multivariate analysis is the use of DIANNA. The following covariates were included in the analysis:

1. Global analysis, low complexity, and high complexity subgroups: number of diagnoses, chief resident’s years of experience, and internal resident’s evaluation.
2. Intermediate complexity subgroup: chief resident’s years of experience and internal resident’s evaluation (excluding the number of diagnoses).

**Table 2:** Percentage of correct differential diagnoses per group.

	DIANNA (n=50)			Control group (n=51)				*P* multivariate
	Mean (SD)	Range	Mean (SD)		Range	*P* univariate		
DD accuracy (%)	79.3 (24)	25-100	70.5 (33)		0-100	0.13	.01	
Low complexity (1-2 DD)	88 (22)	50-100	79.7 (33)		0-100	0.26	.08	
Moderate complexity (3 DD)	68.6 (22)	33.3-100	51.5 (27)		0-100	0.10	.03	
High complexity (4-5 DD)	75 (26)	25-100	60 (30)		20-100	0.31	.91	

### Patients and Physicians’ Satisfaction

The study assessed patient and physician satisfaction with DIANNA, demonstrating high levels of approval from both groups. Patients reported good overall satisfaction with the clarity and relevance of the questions, rating them 3.6 (SD 0.5) on a 5-point scale. In addition, 82% of patients expressed a desire to retain a summary of their DIANNA results, indicating its perceived value as a useful reference for future health care interactions.

Physician feedback further reinforced DIANNA’s clinical utility. The tool was reported to aid in establishing DDs for 26% of RPs, while 82% of residents considered the DDs generated by DIANNA to be comprehensive. In addition, 70% of physicians expressed a willingness to incorporate DIANNA into their future clinical practice, citing its relevance and efficiency in the diagnostic workflow.

These findings suggest that DIANNA not only supports diagnostic accuracy but also enhances physician efficiency and patient engagement. The high satisfaction rates among both patients and physicians indicate that DIANNA has the potential to improve clinical decision-making and contribute to enhanced health care delivery in emergency outpatient settings (Table 3).

**Table 3:** Satisfaction outcomes.

Satisfaction outcomes		Values
AMHTD^a^ suggested DD^b^, mean (SD); range		
	DD accuracy	81.6 (30); 0-100
	Low-complexity index (1-2 DD)	88 (26.1); 0-100
	Moderate-complexity index (3 DD)	84.3 (29.1); 0-100
	High-complexity index (4-5 DD)	52.1 (31.3); 0-100
Patient’s feedback		
	I am able to answer correctly, mean (SD); range	3.9 (0.3); 3-4
	Symptomatology accurately described, mean (SD); range	3.6 (0.5); 2-4
	Length adequate, mean (SD); range	3.6 (0.5); 2-4
	DIANNA^c^ was relevant to my problem, mean (SD); range	3.5 (0.6); 1-4
	Wish to fulfil DIANNA at home, n/N (%)	41/101 (41)
	Wish to keep DIANNA summary, n/N (%)	82/101 (82)
Physician’s feedback		
	AMHTD helps finding DD, n/N (%)	13/50 (26)
	DIANNA DD is exhaustive, n/N (%)	41/50 (82)
	DD clearly presented, mean (SD); range	3 (0.9); 1-4
	DIANNA is pertinent, mean (SD); range	3 (0.7); 2-4
	DIANNA saves time, mean (SD); range	2.4 (0.7); 1-4
	Would you use DIANNA in clinical practice?, n/N (%)	35/50 (70)

^a^AMHTD: automated medical history-taking devices.

^b^DD: differential diagnosis.

^c^DIANNA: diagnosis and anamnesis.

## Discussion

### Principal Findings

Our findings suggest that while DIANNA demonstrated significant potential in enhancing DDs, its effectiveness varied depending on case complexity. The results indicate that DIANNA improved diagnostic accuracy compared with traditional methods, particularly in intermediate-complexity cases, where a statistically significant improvement was observed. This highlights its potential role in enhancing clinical decision-making.

Furthermore, both patients and physicians expressed high levels of satisfaction with DIANNA. A noteworthy percentage of patients indicated a desire to retain a copy of their DIANNA summary for future reference, emphasizing its perceived value in enhancing patient involvement in their own care. However, despite these promising results, further validation is necessary to fully assess DIANNA’s clinical impact, particularly in more complex cases where its effectiveness was less pronounced.

### Comparison to Prior Work

**Figure 2:**
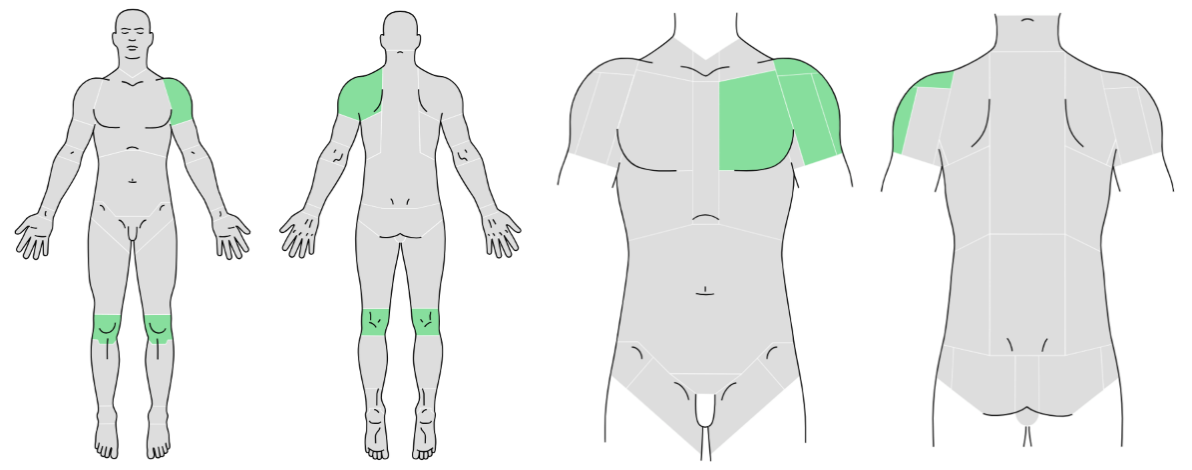
Body pictograms feature in DIANNA.

This study builds upon previous research on automated medical history-taking devices and reinforces the growing recognition of DHHDs as valuable tools in modern health care. The introduction of a body pictogram feature within DIANNA (Figure 2) marks a notable advancement, streamlining the diagnostic process and improving data collection accuracy.

In line with our findings, the existing literature strongly supports the growing role of automated medical history-taking devices in health care. These tools have been shown to:

1. Enhance diagnostic accuracy by reducing physician bias and ensuring structured history-taking [11,12].
2. Improve patient engagement by allowing individuals to actively contribute to their medical history documentation [13].
3. Expedite data collection through structured, AI-assisted questioning that optimizes the consultation process [14].

Moreover, the integration of AI and machine learning into DHHDs represents a major advancement in health care. Unlike standalone AI diagnostic tools, DIANNA functioned as a complementary system, working in conjunction with clinical assessments conducted by chief residents. This hybrid human-AI collaborative model reflects a synergistic approach to health care decision-making, where technology supports but does not replace physician expertise.

### Strengths and Limitations

#### Overview

This study provides valuable insights into the evolution of DHHDs and the continued development of DIANNA. The enhanced version of DIANNA, featuring a body pictogram, represents a significant innovation in optimizing the diagnostic process and improving patient care. By providing structured and standardized history-taking, DIANNA facilitated symptom localization and enhanced DD accuracy, particularly in intermediate-complexity cases.

A major strength of this study is its hybrid human-AI collaboration model, where DIANNA functioned as a decision-support tool rather than an autonomous diagnostic system. This approach ensured that physician expertise remained central to decision-making, with AI augmenting rather than replacing clinical judgment. Furthermore, the study’s real-world application in an emergency outpatient setting highlights its practical usability and feasibility in high-demand clinical environments. Both patients and physicians reported high levels of satisfaction, with many patients expressing interest in retaining a copy of their DIANNA summary for future reference.

Despite these strengths, several limitations must be acknowledged.

#### Single-Center Study

The study was conducted at a single health care center, which may limit the generalizability of its findings to other clinical settings with different patient populations, staffing structures, and medical infrastructures. While efforts were made to contextualize the results by comparing them with existing literature on AI-assisted history-taking tools, the study’s applicability remains largely confined to settings like the University Hospitals Geneva emergency outpatient department. Future studies should address this limitation by conducting multicenter trials across diverse health care settings, ensuring that DIANNA’s effectiveness is evaluated in varied clinical environments.

#### Small Sample Size

With only 101 patients included, the study’s statistical power was limited, increasing the risk of type II errors, where real effects may have gone undetected. The relatively small sample size may also reduce the reliability of subgroup analyses, particularly for high-complexity cases, where larger datasets are necessary to draw meaningful conclusions. While resource constraints and feasibility considerations influenced the study design, expanding sample sizes in future studies will enhance statistical power and improve the ability to detect smaller effect sizes.

#### Pseudorandomized Study Design

Patients were assigned sequentially rather than through pure randomization, introducing the possibility of selection bias. Although pseudorandomization was a practical necessity, baseline characteristics were carefully assessed to ensure comparability between the control and intervention groups. However, some systematic differences may have persisted, potentially influencing the results. Future research should use fully randomized controlled trials to provide stronger causal inferences about DIANNA’s impact and further eliminate potential bias in patient allocation.

#### Impact of COVID-19 on Recruitment

The study’s recruitment process was affected by interruptions due to COVID-19–related restrictions, leading to variability in patient selection and flow. These disruptions may have introduced bias by altering the demographics and clinical characteristics of participants, as health care–seeking behaviors changed during the pandemic. Efforts were made to resume recruitment as soon as feasible while maintaining consistent eligibility criteria, but the possibility of pandemic-related effects on diagnostic patterns cannot be entirely excluded. Future studies should aim for uninterrupted recruitment periods or consider external factors influencing patient presentation when analyzing results.

#### Short-Term Outcomes Without Long-Term Follow-Up

The study focused primarily on immediate diagnostic accuracy and consultation efficiency, without evaluating long-term patient outcomes such as treatment adherence, health care usage, or sustained clinical effectiveness. While this short-term focus was an intentional choice due to feasibility constraints, it limits the study’s ability to determine whether AI-assisted history-taking translates into improved long-term patient care. Future research should incorporate longitudinal follow-up to assess whether DIANNA’s benefits persist over time and contribute to better clinical outcomes and patient safety.

#### Use of a SP’s Diagnosis as the Gold Standard

The study relied on a single SP’s diagnosis as the gold standard for comparison, which introduces potential subjectivity in diagnostic validation. Although assessments were cross-referenced with clinical guidelines, interphysician variability could still influence diagnostic accuracy measurements. This potential bias highlights the need for multiple independent reviewers or consensus panels in future studies to improve reliability and objectivity in evaluating diagnostic accuracy.

#### Potential Learning Effect Among Physicians

Physicians using DIANNA may have become more familiar with its output over time, leading to improved diagnostic performance as they adapted to the tool. This learning effect could have inflated DIANNA’s observed effectiveness, particularly in cases where physicians refined their ability to interpret and integrate AI-generated suggestions. Although pseudorandomization helped minimize bias, future studies should consider using crossover designs, where the same group of physicians rotates between using DIANNA and standard history-taking methods, to control for potential experience-related improvements.

### Future Directions

Future research should focus on large-scale, multicenter trials to enhance the generalizability of DHHDs such as DIANNA across diverse health care settings. Beyond immediate diagnostic accuracy, studies should assess long-term patient outcomes, including treatment adherence and clinical effectiveness. Seamless integration with electronic health records will be essential for optimizing usability and supporting real-time decision-making.

Data security, privacy, and regulatory compliance must also be prioritized to ensure ethical implementation. Comparative studies between different DHHDs will help establish best practices and refine standardized guidelines. As AI technology continues to advance, DHHDs have the potential to improve diagnostic precision, enhance patient engagement, and streamline clinical workflows.

The future of health care will likely be shaped by hybrid models where AI supports, rather than replaces, physician expertise. Ensuring a balanced collaboration between AI-driven tools and clinicians will be key to maximizing their impact on patient care.

### Conclusion

The findings of this study reinforce the transformative potential of DHHDs such as DIANNA in clinical practice. While AI-driven medical history tools are still evolving, their integration into hybrid health care models holds promise for enhancing diagnostic accuracy, improving efficiency, and strengthening patient-centered care. Future research should focus on longitudinal validation, multicenter trials, and integration with electronic health records to optimize the clinical utility of AI-assisted history-taking tools.

## Abbreviations

AI: artificial intelligence
DD: differential diagnosis
DHHD: digital health history device
DIANNA: diagnosis and anamnesis
RP: resident physician
SP: senior physician

## Data Availability

The datasets generated or analyzed during this study are available from the corresponding author on reasonable request to Adrien.schwitzguebel@gmail.com.
